# Unraveling the potential of graphene quantum dots against *Mycobacterium tuberculosis* infection

**DOI:** 10.3389/fmicb.2024.1395815

**Published:** 2024-05-07

**Authors:** Giulia Santarelli, Giordano Perini, Alessandro Salustri, Ivana Palucci, Roberto Rosato, Valentina Palmieri, Camilla Iacovelli, Silvia Bellesi, Michela Sali, Maurizio Sanguinetti, Marco De Spirito, Massimiliano Papi, Giovanni Delogu, Flavio De Maio

**Affiliations:** ^1^Dipartimento di Scienze Biotecnologiche di Base, Cliniche Intensivologiche e Perioperatorie-Sezione di Microbiologia, Università Cattolica del Sacro Cuore, Rome, Italy; ^2^Dipartimento di Neuroscienze, Università Cattolica del Sacro Cuore, Rome, Italy; ^3^Fondazione Policlinico Universitario “A. Gemelli”, IRCCS, Rome, Italy; ^4^Istituto dei Sistemi Complessi, CNR, Rome, Italy; ^5^Mater Olbia Hospital, Olbia, Italy

**Keywords:** Graphene Quantum Dots (GQDs), *Mycobacterium tuberculosis (Mtb)*, anti-tuberculosis therapy, drug resistance, nanoparticle adjuvants

## Abstract

**Introduction:**

The emergence of drug-resistant *Mycobacterium tuberculosis (Mtb)* strains has underscored the urgent need for novel therapeutic approaches. Carbon-based nanomaterials, such as graphene oxide (GO), have shown potential in anti-TB activities but suffer from significant toxicity issues.

**Methods:**

This study explores the anti-TB potential of differently functionalized graphene quantum dots (GQDs) – non-functionalized, L-GQDs, aminated (NH_2_-GQDs), and carboxylated (COOH-GQDs) – alone and in combination with standard TB drugs (isoniazid, amikacin, and linezolid). Their effects were assessed in both axenic cultures and *in vitro* infection models.

**Results:**

GQDs alone did not demonstrate direct mycobactericidal effects nor trapping activity. However, the combination of NH_2_-GQDs with amikacin significantly reduced CFUs in *in vitro* models. NH_2_-GQDs and COOH-GQDs also enhanced the antimicrobial activity of amikacin in infected macrophages, although L-GQDs and COOH-GQDs alone showed no significant activity.

**Discussion:**

The results suggest that specific types of GQDs, particularly NH_2_-GQDs, can enhance the efficacy of existing anti-TB drugs. These nanoparticles might serve as effective adjuvants in anti-TB therapy by boosting drug performance and reducing bacterial counts in host cells, highlighting their potential as part of advanced drug delivery systems in tuberculosis treatment. Further investigations are needed to better understand their mechanisms and optimize their use in clinical settings.

## Introduction

Tuberculosis (TB) remains a major global health concern, being one of the leading causes of death from a single infectious agent (World Health Organization, 2022). The disease is cured with a multidrug pharmacological treatment, consisting of daily dosing of a four-drug regimen, for a period ranging from 4 to 8 months or longer (Timothy, 2022). The emergence of multidrug resistant and extensively drug resistant *Mycobacterium tuberculosis* (*Mtb*) strains makes the identification of novel potential treatment strategies a priority. The search for new solutions includes the investigation of both novel agents and repurposing approved drugs with potential anti-mycobacterial effects (Palucci and Delogu, 2018; Battah et al., 2019; De Maio et al., 2022).

Carbon-based Nanomaterials (CNM) treatments, particularly Graphene-Oxide (GO) have showed a promising activity against mycobacteria (De Maio et al., 2019). Even though GO did not show a direct bactericidal activity, it was able to entrap mycobacteria in a net thus interfering with the normal infection of macrophages (De Maio et al., 2019). Furthermore, co-administration of GO with linezolid, a second line anti-TB drug, resulted in a synergistic anti-*Mtb* effect, due also to the increase of reactive oxygen species (ROS) production (De Maio et al., 2020). However, interaction of GO sheets with isoniazid or amikacin interfered and hindered antibiotic activity (De Maio et al., 2020). Moreover, when GO was used in a *Mtb* infection model based on peripheral blood mononuclear cells, we observed a failure in controlling mycobacterial replication, largely due to the toxicity of GO against monocytes and CD4 T cells (Salustri et al., 2023).

Among CNMs with size less than 100 nm, Graphene quantum dots (GQDs) and carbon quantum dots (CQDs) represent two distinct classes of nanomaterials with unique structural and functional attributes. GQDs are monolayer or few-layer structures filled with various oxygen-containing functional groups like hydroxyl, epoxy, and carboxyl. These functional groups not only render GQDs hydrophilic and biocompatible but also facilitate functionalization, enabling specific interactions with biological systems. Functionalization of GQDs with amino (–NH_2_) or carboxyl (–COOH) groups further differentiates their biological and chemical properties. Amino-functionalized GQDs (NH_2_-GQDs) exhibit enhanced interaction with biological systems, as the amino groups can facilitate cell penetration and targeting, making them suitable for applications in bioimaging and drug delivery. The amine groups can form amide bonds with biomolecules, enhancing the stability and biocompatibility of the complexes formed. On the other hand, Carboxylated GQDs (COOH-GQDs) have strong acidic properties and can interact with positive charges on the surface of proteins and other biomolecules. This interaction capability makes COOH-GQDs particularly useful in biosensing and molecular recognition applications, where selective binding to target molecules is crucial. GQDs’ surface chemistry and their functionalization confer them higher specificity in biological and chemical interactions. That makes them of particular interest in nanotechnology (Henna and Pramod, 2020). For these reasons, recently, the interest in GQDs has widely spread toward different branches of biology and medicine, making them excellent candidates for use in bioimaging, drug delivery and in theranostic applications (Perini et al., 2020).

Studies on the graphene-family of nanomaterials led to the development by Ponomarenko and Geim in 2008 of GQDs, that were considered more suitable for biological applications than any other quantum dots (Henna and Pramod, 2020). Indeed, GQDs are carbon-based zero-dimensional fluorescent nanomaterials with a graphene lattice inside, show great solubility, have a size less than 20 nm (maximum size of 60 nm) and can easily penetrate through biological membranes. Additionally, GQDs suspension is very stable in a high electrolyte concentration and at low pH solutions (Henna and Pramod, 2020).

Unlike graphene and similarly to fullerenes (C60), GQDs in suspension can generate reactive oxygen species (ROS) upon photoexcitation (Christensen et al., 2011). Therefore, GQDs are potential candidates for photodynamic therapy, in which the light-excited compound kills cells by ROS generated through energy or electron transfer to molecular oxygen (Robertson et al., 2009). Photodynamic therapy can also target microbial pathogens, including bacteria, which is becoming increasingly relevant considering the emerging antibiotic resistance and consequent reduction in effectiveness of conventional therapies. While most carbon-based nanomaterials, including fullerenes, carbon nanotubes and graphene display antibacterial properties (Liu et al., 2011), the effects of GQDs on bacteria have not been investigated so far. It has been shown that electrochemically produced GQDs generate reactive oxygen species when photoexcited (470 nm, 1 W), and can kill two strains of pathogenic bacteria as methicillin-resistant *Staphylococcus aureus* and *Escherichia coli* (Ristic et al., 2014). Moreover, small-sized GQDs may assist in the drug delivery process thanks to their ability to permeate vertically the lipid membrane on a nanosecond timescale, without mechanically causing damage (Xue et al., 2019).

In this work, our interest was to investigate the role of GQDs as antimycobacterial compounds alone or in combination with anti-*Mtb* drugs in *in vitro* and in *ex vivo* infection models.

## Results

### Characterization of GQDs

**Figure 1:**
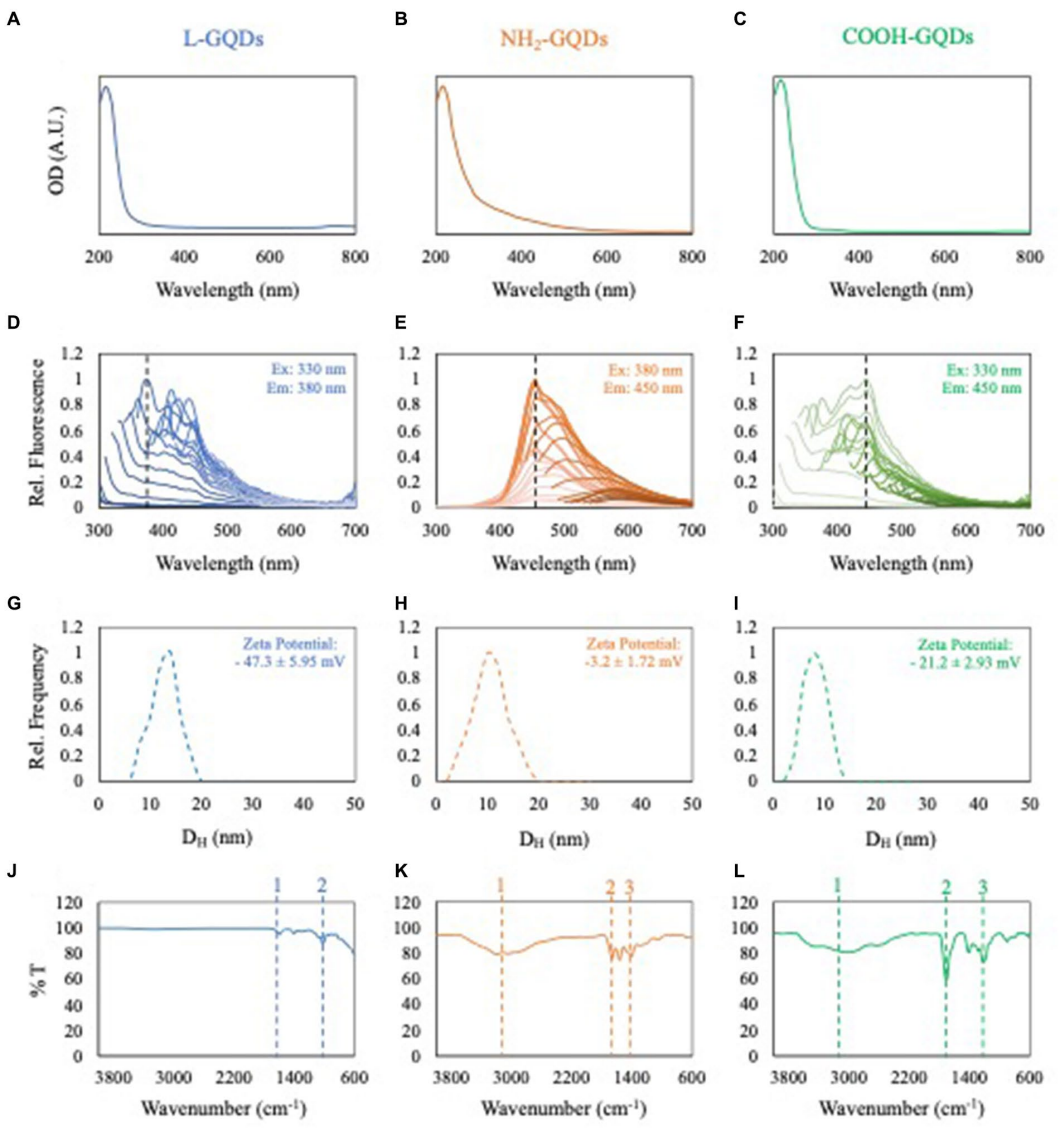
Characterization of GQDs. Optical density of L-GQDs, NH2-GQDs and COOH-GQDs, respectively **(A–C)**. Fluorescence spectra of L-GQDs, NH2-GQDs and COOH-GQDs by exciting from 250 to 520 nm and recording emission from 300 to 700 nm. Data were normalized to the highest recorded fluorescence intensity for each GQD **(D–F)**. Dynamic light scattering and zeta potential of L-GQDs, NH2-GQDs and COOH-GQDs, respectively **(G–I)**. IR spectra of L-GQDs, NH2-GQDs and COOH-GQDs showing their chemical composition **(J–L)**.

GQDs underwent comprehensive characterization encompassing optical density, fluorescence intensity, dynamic light scattering (DLS), zeta potential, and attenuated total reflection-Fourier transform infrared spectroscopy (FTIR). The outcomes are depicted in Figure 1. Both L-GQDs and COOH-GQDs exhibited an absorption shoulder at 350 nm, whereas NH_2_-GQDs demonstrated a shifted absorption peak toward 400 nm (Figures 1A–C). Fluorescence intensity spectra were recorded by exciting GQDs from 250 to 520 nm and measuring emission from 300 to 700 nm with a 10 nm step size. Data normalization was performed relative to the highest recorded emission for each GQD. L-GQDs manifested an emission peak at 380 nm when excited at 330 nm (Figure 1D). NH_2_-GQDs exhibited maximum emission at 450 nm when excited at 380 nm (Figure 1E). COOH-GQDs displayed an emission peak at 450 nm when excited at 330 nm (Figure 1F). DLS and zeta potential analyses were conducted, indicating a consistent size distribution with a hydrodynamic radius peaked below 10 nm for all particles (Figures 1G–I). Zeta potential measurements revealed distinct surface net charges for the three chemically functionalized nanoparticles. L-GQDs and COOH-GQDs exhibited more negative charges, as anticipated (Perini et al., 2020), compared to NH_2_-GQDs (Figures 1G–I). L-GQDs possessed a surface charge of −47.3 ± 5.95 mV, while COOH-GQDs showed a less negative charge of −21.2 ± 2.93 mV. Due to the presence of amino groups, NH_2_-GQDs exhibited a zeta potential of −3.2 ± 1.72 mV. Surface chemical functionalization of GQDs was confirmed and examined through FTIR spectroscopy. The IR spectra of L-GQDs revealed two bands in the fingerprint region typical of the C=O bond (bands 1 and 2, Figure 1J) (Perini et al., 2023). NH_2_-GQDs displayed a broad band around 3,000 cm^−1^, corresponding to the N–H stretching vibration of amine groups (Figure 1K, peak 1). Peaks in the fingerprint region indicated the presence of amide I and amide II, while a broader band at around 1,400 cm^−1^ indicated C-N absorption (Figure 1K, peaks 2 and 3, respectively). The IR spectra of COOH-GQDs exhibited characteristic frequencies of the carboxylic group, a broad band between 3,500–2,500 cm^−1^ for the O-H stretch (Figure 1L, peak 1), and a prominent band at 1705 cm^−1^ for the C=O stretch (Figure 1L, peak 2).

#### NH_2_-GQDs enhances AMK activity against *Mtb* but does not exert any direct antimycobacterial effect alone

**Figure 2:**
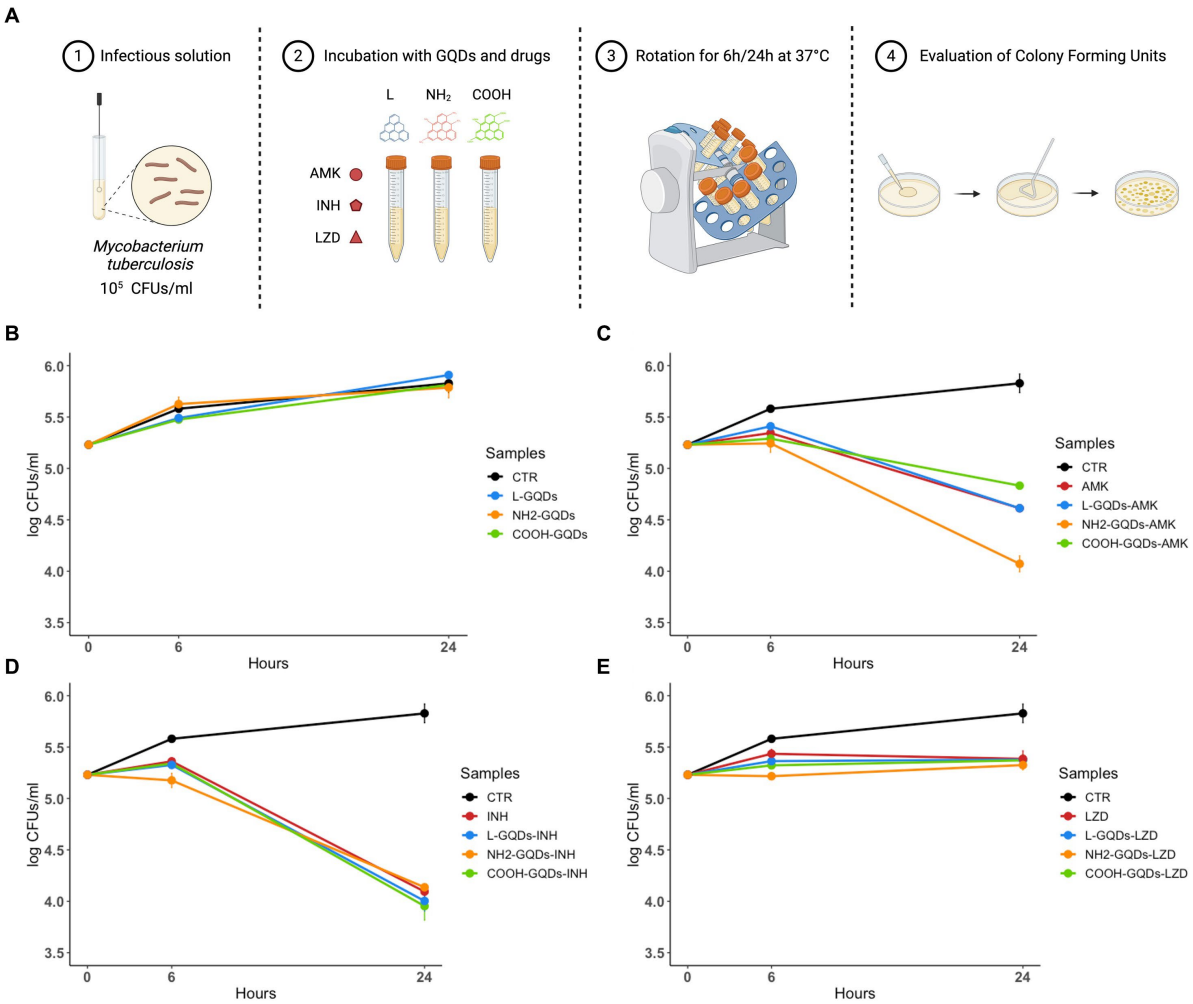
GQDs and GQDs-drugs formulations activity on *Mtb* H37Rv. Schematic representation depicts the experimental model used to assay Graphene Quantum Dots (GQDs) and GQDs – anti mycobacterial drugs administration on *Mycobacterium tuberculosis* (*Mtb*) **(A)**. A suspension of *Mtb* H37Rv at the final concentration of 10^5^ CFUs/ml was incubated with 50 μg/mL GQDs (L-GQDs, NH_2_-GQDs and COOH-GQDs) alone or in combination with amikacin (AMK), isoniazid (INH) and linezolid (LZD) at minimal inhibitory concentrations (MICs). Untreated *Mtb* culture was used as control (CTR) **(B–E)**. Colonies forming units were assessed 6 h (h) and 24 h post incubation and represented in log10 scale.

To assess the direct antimycobacterial activity of differentially functionalized GQDs (L-GQDs, NH_2_-GQDs and COOH-GQDs), each nanomaterial was administered alone or in combination with INH, AMK and LZD (at the respective MIC) to *Mtb* H37Rv culture, and antimycobacterial activity was measured 6 and 24 h later (Figure 2A). GQDs alone were not able to reduce mycobacterial counts (*p* > 0.05) suggesting that the trapping effect, mainly dependent on CNMs destabilization in biological fluids, was not achieved for the smaller GQDs (Figure 2B). Interestingly, both INH and LZD co-administrated with GQDs did not enhance their efficacy 24 h p.i. (*p* > 0.05), while a slight improvement of the AMK activity was observed (Figures 2C–E). Hence, co-administration of AMK with NH_2_-GQDs significantly reduced CFUs in comparison with AMK alone or AMK in combination with L-GQDs and COOH-GQDs reaching levels comparable to INH treatment (reduction: log1.13, *p* value <0.001).

**Figure 3:**
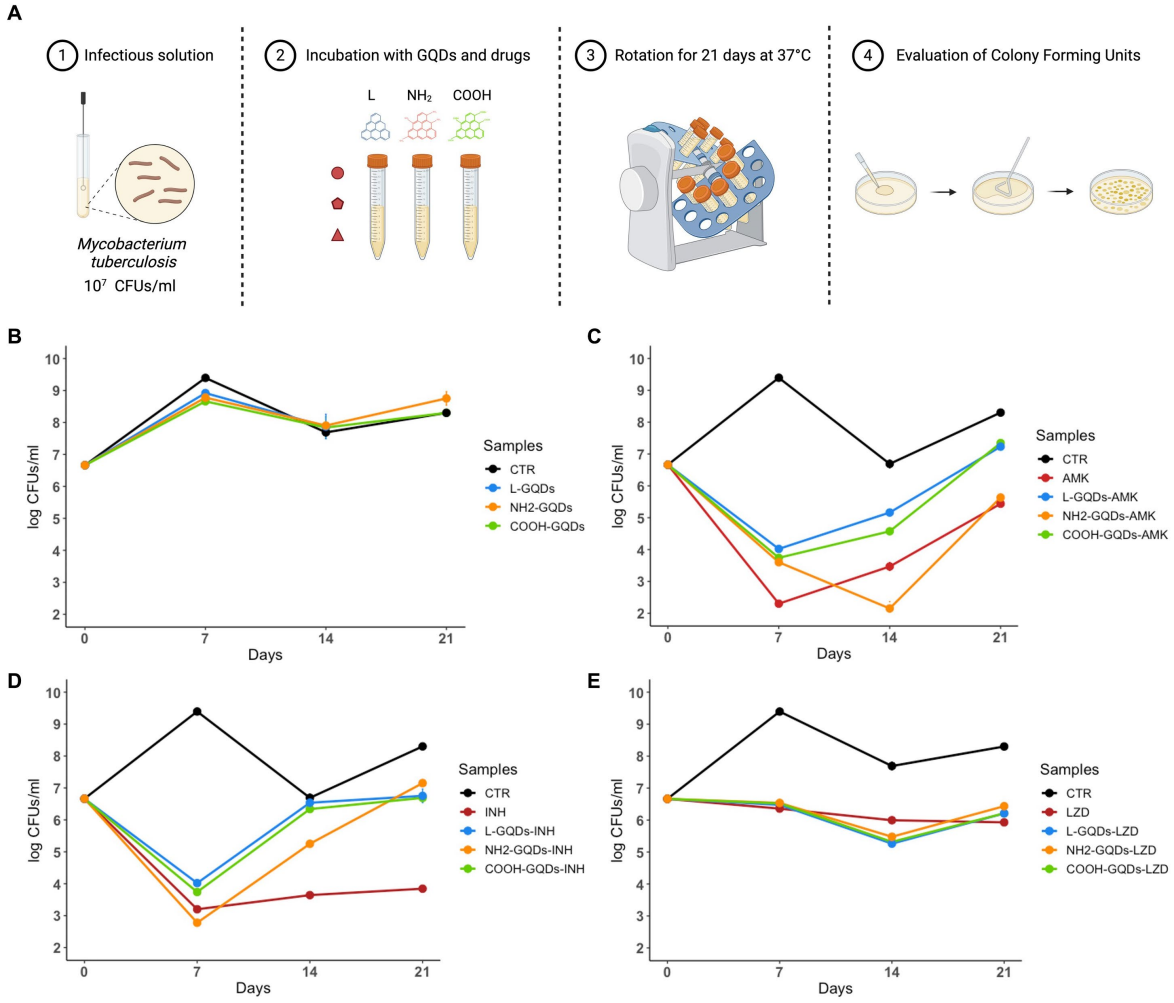
GQDs and GQDs-drugs formulations activity on exponentially growing cultures of *Mtb* H37Rv. Schematic representation depicts the experimental model used to assay Graphene Quantum Dots (GQDs) and GQDs anti mycobacterial drugs administration on exponentially growing *Mycobacterium tuberculosis* (*Mtb*) **(A)**. A solution of *Mtb* H37Rv at the final concentration of 10^7^ CFUs/ml was incubated with 50 μg/mL GQDs (L-GQDs, NH_2_-GQDs and COOH-GQDs) alone or administrated with amikacin (AMK), isoniazid (INH) and linezolid (LZD) at minimal inhibitory concentrations (MICs). Untreated *Mtb* culture was used as control (CTR) **(B–E)**. Colonies forming units were measured at 7, 14, and 21 days and represented in log10 scale.

GQDs typically interact with cellular membrane destabilizing the lipidic shift or altering surface net charge without modifying cell uptake (Wang et al., 2015; Zhang et al., 2022). Taking into account this property, we assayed the GQDs direct activity against exponentially growing mycobacterial cultures, by incubating nanoparticles alone or co-administrated with AMK, INH and LZD up to 21 days (Vilcheze et al., 2017). CFUs were determined at 0, 7, 14 and 21 days by plating serial dilutions of the suspensions (Figure 3A).

Interestingly, neither GQDs alone nor GQDs combined with INH and LZD exhibited a significant antimicrobial effect compared to INH and LZD treatment (Figures 3B,D,E). Conversely, NH_2_-GQDs – AMK administration appeared the only combination to slightly reduce CFUs, maintaining at 21 days similar levels to AMK alone (*p* > 0.05). While 7 days p.i., AMK alone appeared significantly more efficient than GQDs-AMK combinations, NH_2_-GQDs – AMK administration determined a major CFUs decreasing at 14 days (reduction: log1.61, *p* < 0.001) (Figure 3C).

Taken together these results indicate that GQDs do not exert antimycobacterial activity except for the combined use of NH_2_- NH_2_-GQDs that enhanced the antimycobacterial activity of AMK when co-administered. Furthermore, the major aminated GQDs-AMK activity, compared to material or drug alone, appears dependent on the mycobacterial count.

#### NH_2_-GQDs – AMK co-administration improves antimycobacterial drug activity during macrophages infection

Given the proven efficacy of GQDs to perturb the cell’s lipidic membrane and increase its permeability (Perini et al., 2020), we tested the GQDs-drugs activity in *in vitro* models of *Mtb* infection based on murine macrophages (J774 A.1 cell line).

**Figure 4:**
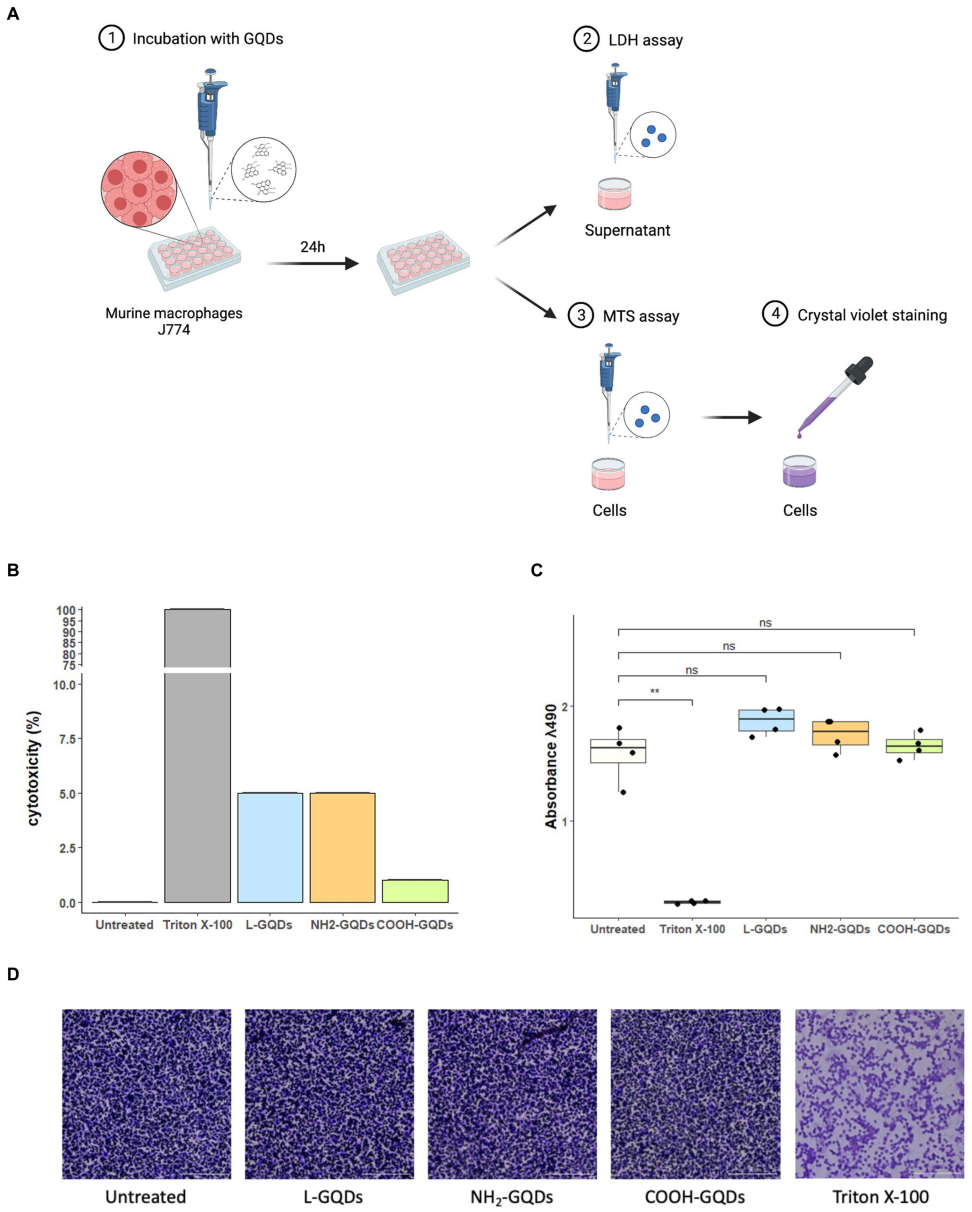
Assessment of GQDs’ cytotoxicity on murine macrophages. Murine macrophages (J774) were seeded in a 96 well plate. When 90% cell monolayer was reached, cells were treated with 50 μg/mL GQDs. Untreated cells and cells treated with 2% Triton X-100 were used as negative and positive controls, respectively **(A)**. One day post-incubation, Lactate DeHydrogenase (LDH) was measured in culture supernatant and cytotoxicity was expressed as percentage of LDH release normalized on negative and positive controls **(B)**. The metabolic activity was evaluated following MTS assay, and the images of cellular monolayer’s integrity were acquired by using cytation instrument after crystal violet staining **(C,D)**.

Biocompatibility of GQDs on eukaryotic cells was measured by incubating J774 with L-GQDs, NH_2_-GQDs and COOH-GQDs, as previously described (Perini et al., 2023). Following 24 h of incubation, we did not observe differences between the functionalized GQDs in terms of Lactate DeHydrogenase (LDH) release, metabolic activity (MTS assay) and monolayer integrity (Cristal violet staining) (Figure 4). The percentage of cytotoxicity measured by the LDH release revealed a cytotoxicity of functionalized GQDS comparable to that of untreated cells (0.05, 0.05 and 0.01%, for L-GQDs, NH_2_-GQDs and COOH-GQDs, respectively). Furthermore, MTS assay results showed that the metabolic activity of macrophages was not affected by GQDs, suggesting that neither cellular activation nor cellular death were induced by nanomaterials. As expected, treatment with Triton X-100 significantly decreased metabolic activity as compared to control (*p* value <0.01). MTS assay was also performed on hepatic immortalized cells (HepG2) and renal immortalized cells (Vero) confirming GQDs’ biocompatibility (Supplementary Figure 1).

**Figure 5:**
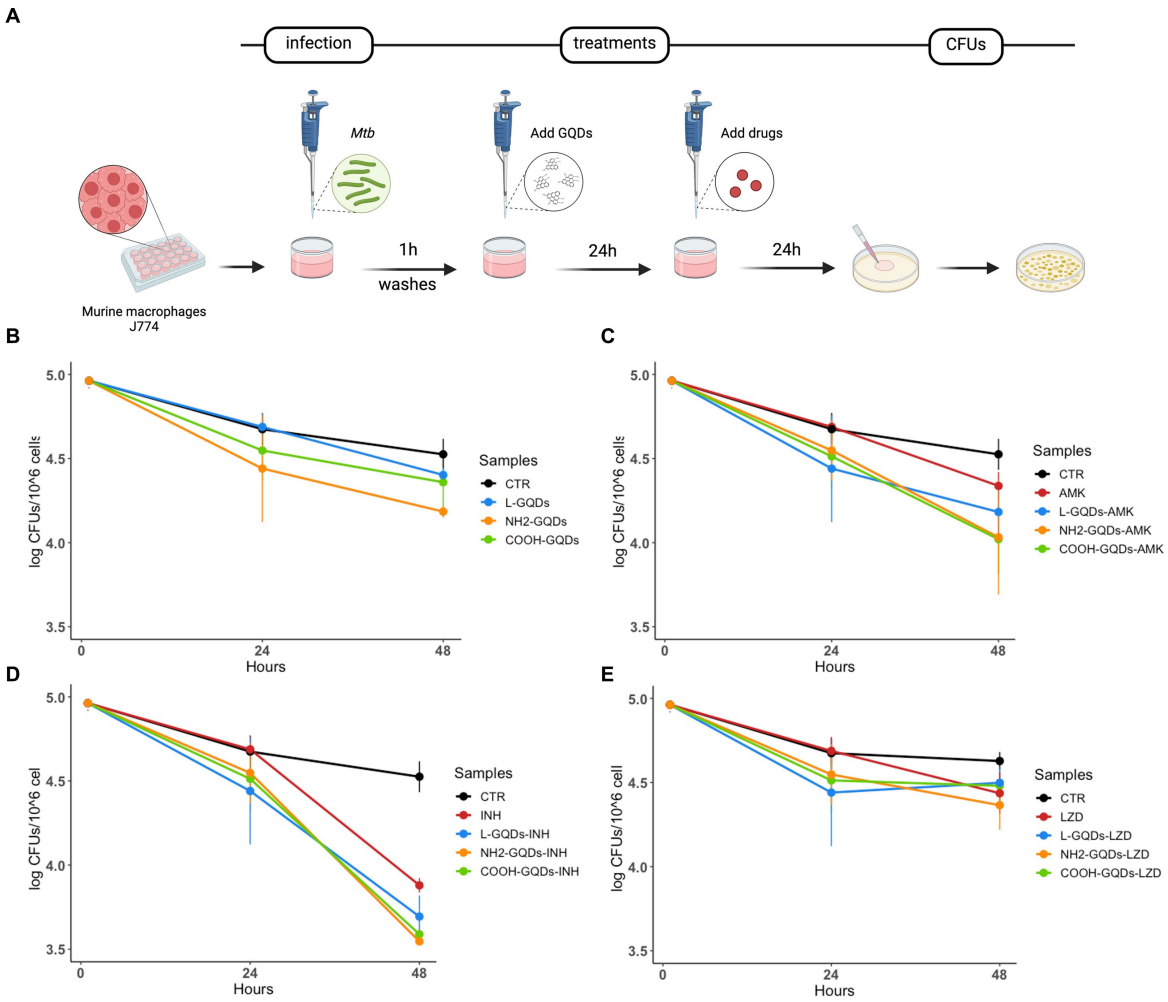
GQDs-drugs effects during *Mtb* infection on differentiated macrophages. Murine macrophages (J774) were infected with *Mycobacterium tuberculosis* (*Mtb*) H37Rv (MOI = 1:1). One hour after infection, cells were washed and treated with 50 μg/mL GQDs (L-GQDs, NH_2_-GQDs and COOH-GQDs). The next day, the medium was removed and new fresh medium containing amikacin (AMK), isoniazid (INH) and linezolid (LZD) at minimal inhibitory concentrations (MICs) was added **(A)**. Colony forming units (CFUs) were evaluated 24 h post treatment **(B–E)** and represented in log10 scale.

J774 were infected with *Mtb* for 1 h (MOI of 1:1), then extracellular bacteria were removed, and cells treated with GQDs for 1 day. Finally, INH, AMK and LZD were administrated at the indicated concentrations. Cells were incubated in standard atmosphere conditions for additional 24 h when CFUs were assessed by plating serial dilutions of each condition (Figure 5A). No significant anti-mycobacterial activity was observed at 24 h p.i. for macrophages treated with L-GQDs and COOH-GQDs alone, while treatment with NH_2_-GQDs resulted in a reduction of CFUs (*p* = 0.014) when compared to untreated cells (Figure 5B). Additionally, NH_2_-GQDs and COOH-GQDs significantly enhanced AMK activity at 48 h p.i. in comparison with untreated macrophages, by promoting a reduction of 1.12 Log CFU (*p* < 0.01) and 1.07 LogCFU, respectively, with the latter difference not reaching statistically significance compared to macrophages treated with AMK alone (Figure 5C). Conversely, all tested GQDs did not enhance INH and LZD activities (Figures 5D,E). These results show that NH_2_-GQDs slightly improves macrophagic response against mycobacterial replication and that NH_2_-GQDs and COOH-GQDs enhance AMK antimycobacterial activity when macrophages are pre-treated with these nanomaterials.

#### GQDs does not control *Mtb* replication in hPBMCs

**Figure 6:**
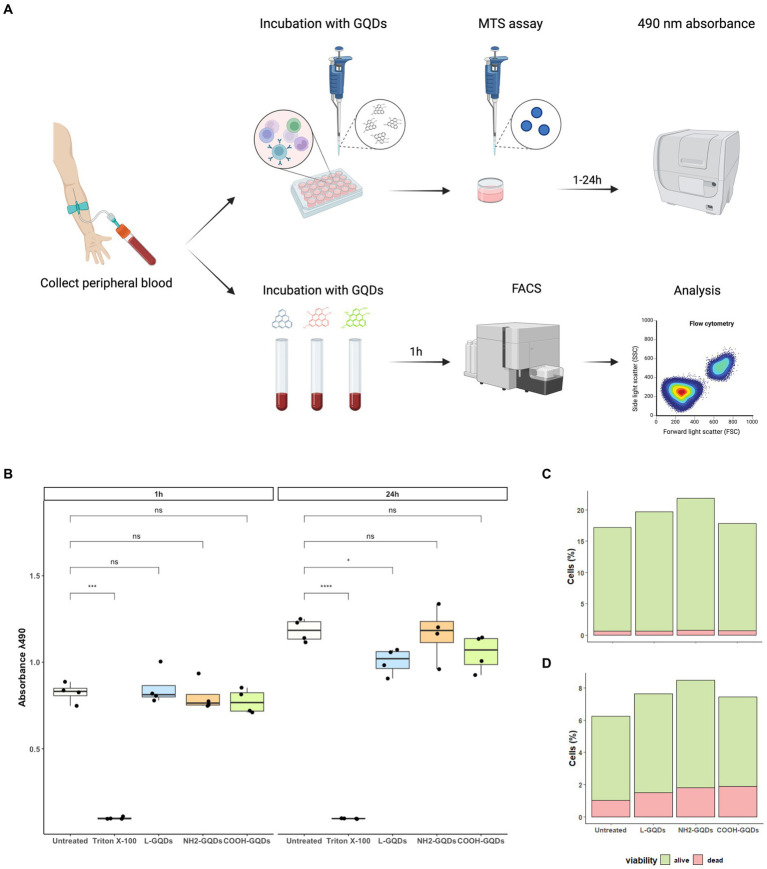
Assessment of GQDs’ cytotoxicity on hPBMCs and on whole blood cells. Human Peripheral Blood Mononuclear Cells (hPBMCs) were isolated from healthy donors and treated with GQDs for 1 and 24 h **(A)**. After incubation, MTS assay was performed to evaluate cellular metabolic activity, using untreated cells and cells treated with 2% Triton X-100 as negative and positive controls, respectively **(B)**. Furthermore, the peripheral whole blood was incubated with 50 μg/mL GQDs (L-GQDs, NH_2_-GQDs and COOH-GQDs) for 1 h at 37°C. Cells viability was measured through flow cytometry analysis by using 7AAD PC5.5-A staining and Side Scatter (SSC-A) gating. Viability of lymphocytes **(C)** and monocytes **(D)** was quantitatively assessed after treatment with nanomaterials.

We have recently demonstrated that GO exerts a generalized toxic effect on human PBMCs (Salustri et al., 2023), hence we aimed to investigate whether GQDs have any toxicity in similar settings. We assessed cellular viability 1 h post treatment measuring 7AAD PC5.5-A signal (7AAD PC5.5-A binds DNA with high affinity and it is efficiently excluded by viable cells resulting an optimal marker for dead cells which have damaged nuclear membranes). To corroborate FACS analysis, MTS assay was performed on hPBMCs stimulated with GQDs for 1 h and 24 h (Figure 6A). GQDs did not show any toxicity on hPBMCs at both 1 h and 24 h post treatment (Figure 6B). Among the leukocytes, both lymphocytes (CD3 positive cells) and monocytes (CD64 positive population) were not affected by GQDs (Figures 6C,D) and the percentage of living cells was comparable to that measured in the untreated samples (20 and 8%, respectively), while less than 1–2% appeared dead after treatment. These results suggest that no cytotoxicity was induced by GQDs on hPBMCs.

**Figure 7:**
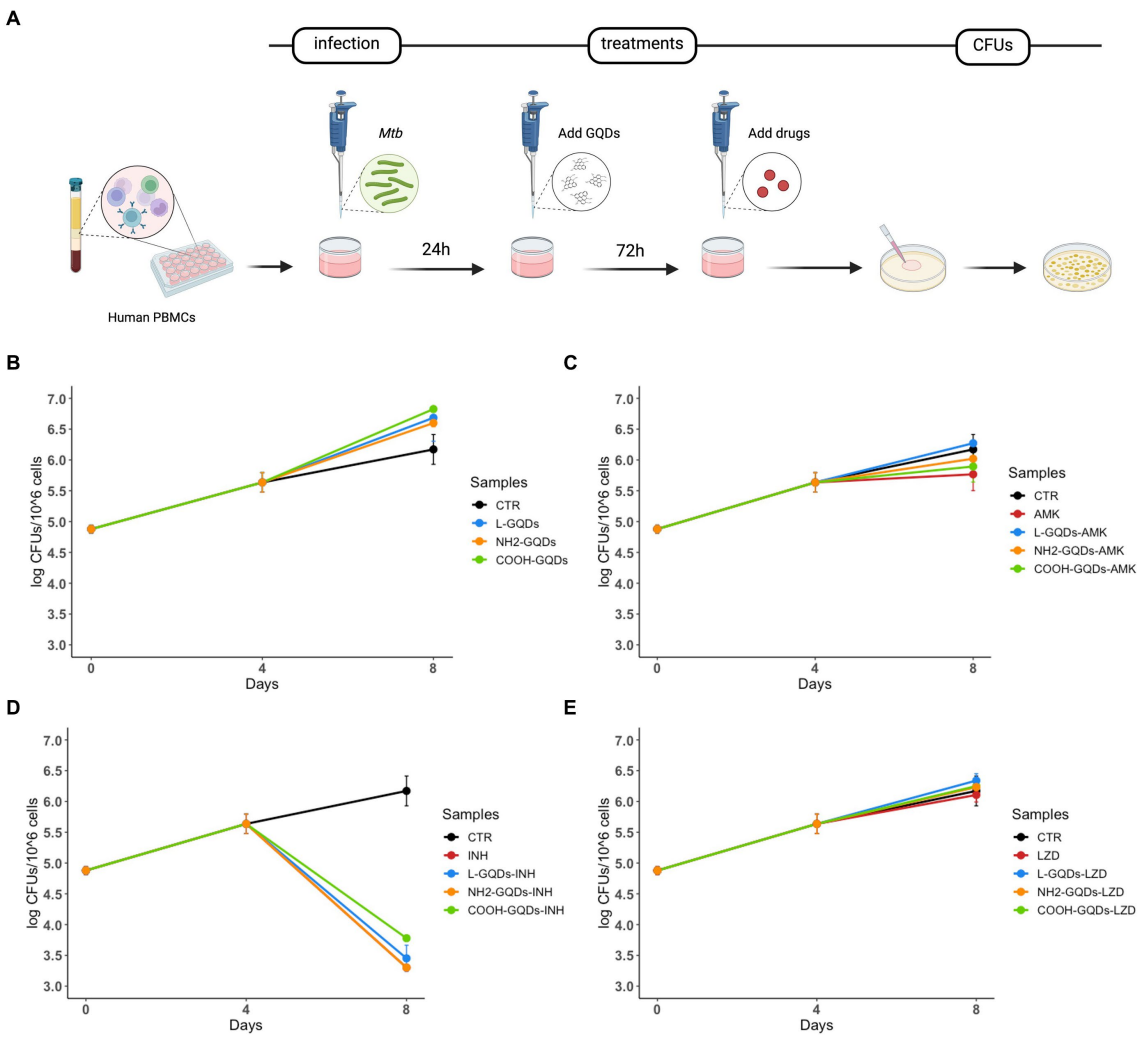
GQDs-drugs cytotoxicity effects on *Mtb ex vivo* hPBMCs infection model. Human peripheral blood mononuclear cells (hPBMCs), isolated from healthy donors, were infected with *Mycobacterium tuberculosis* (*Mtb*) H37Rv (MOI of 1 respect to monocytes, corresponding to ~5% of the hPBMCs, ~ 6×10^4^ cells). One day after infection, cells were treated with 50 μg/mL GQDs (L-GQDs, NH_2_-GQDs and COOH-GQDs) **(A)**. Four days post infection, amikacin (AMK), isoniazid (INH) and linezolid (LZD) at minimal inhibitory concentrations (MICs) were administrated. Colony forming units (CFUs) were evaluated at 4- and 8-days post infection and represented in log10 scale **(B–E)**.

To investigate whether GQDs were able to affect *Mtb* viability in this model of infection, hPBMCs isolated from healthy donors were infected with *Mtb* H37Rv, treated with GQDs 24 h p.i., before AMK, INH and LZD administration 3 days later (Figure 7A). Bacterial survival was finally evaluated 8 days post-infection. As shown in Figure 7B, GQDs alone did not affect mycobacterial replication compared to the untreated hPBMCs. Moreover, no minor effects were observed when antibiotics were administrated together on GQDs pre-treated cells (*p* > 0.05) (Figures 7C–E).

These results corroborated the absence of GQDs toxicity on hPBMCs and highlight, at least in these formulations and using this experimental setting, no evident activity against *Mtb* infection. Moreover, non-functionalized L-GQDs may also have a opposite effect in controlling mycobacterial replication.

## Discussion

The last 10 years have seen a growing interest in the medical application of CNM and particularly of GO, mostly thanks to its biocompatibility, antimicrobial properties and, regarding *Mtb* infection, its ability to accumulate in the lungs (Zhang et al., 2011; Park et al., 2015; Palmieri et al., 2017; De Maio et al., 2019). We have demonstrated that GO can entrap extracellular mycobacteria (De Maio et al., 2019) and, depending on its surface-exposed functional groups, enhances the anti-*Mtb* activity of LZD, but not of INH and AMK (De Maio et al., 2020). However, GO exerts a toxic effect on immune cells reducing monocytes viability and prompting an early activation of CD4 lymphocytes (Salustri et al., 2023).

Among CNMs, GQDs raised interest in biomedical applications and may provide effective and less toxic scaffolds against bacterial infections, including those caused by mycobacteria. GQDs are readily cleared from the body, a key feature for their biocompatibility. *In vivo* biodistribution experiments of GQDs revealed no accumulation in main organs of mice and fast clearance through kidney. Furthermore, multi-dosing administration of GQDs did not show any influence on mice owing to its small size, while GO appeared toxic, even caused mice death (Chong et al., 2014).

The uptake and clearance of GQDs appear both cell type dependent and GQDs surface charge dependent. GQDs with similar sizes (~5–8 nm) but carrying different surface charges (cationic and anionic) show differences in their uptake and clearance in melanoma cells (Ramana et al., 2021). Both GQDs exhibited comparable biocompatibility, with the cationic GQDs showing a faster and higher uptake compared to anionic GQDs. Consequently, a relatively rapid clearance was observed in cells treated with anionic GQDs compared to those treated with cationic GQDs (Ramana et al., 2021). Based on these observations, we set up a series of experiments to investigate the role of differently functionalized GQDs against *Mtb* infection.

GQDs significantly improve GO aqueous stability while maintaining wide chemical adaptability and high adsorption capacity (Chong et al., 2014). Our results highlight that GQDs alone do not directly affect mycobacterial viability. Nevertheless, GQDs show direct effect when co-administrated with anti-TB drugs; in particular, NH_2_-GQDs – AMK and COOH-GQDs – AMK combinations significantly reduce CFUs in comparison with AMK alone or its combination with not functionalized L-GQDs. Importantly, combining GQDs with AMK or INH does not promote precipitation of the nanomaterial (data not shown), allowing the conservation of the drug activity, different to what observed for GO (De Maio et al., 2020). While aqueous solubility and stability of GO are mainly dependent on the oxygen-rich functional groups at the sheets’ edges, this problem is overcome for GQDs due their smaller size. Unfortunately, their size prevents the aggregation in biological fluids observed for GO, thus mycobacteria entrapment in nanomaterial-like nets.

Clearance of GQDs, and more in general of nanomaterials, is executed by macrophages which are responsible for recognizing and ingesting these particles (Dobrovolskaia et al., 2017). Macrophages also represents the first line of host defense and trigger the early immune response against mycobacteria (Ahmad et al., 2022). Previous experiments demonstrated that GQDs were engulfed in the murine J774 macrophage cell lines thus stimulating intracellular ROS production that interferes with macrophages proliferation (Qu et al., 2013). J774 is a globally used model to assay *Mtb* infection that we adopted for our experiments. Contrary to what observed by Qu and colleagues, we did not observe any toxicity following GQDs treatments, confirming that surface modification ensured the function and the safety of the GQDs. Moreover, GQDs are increasingly recognized as anti-cancer drug carriers in many experiments due to their ability to facilitate the permeation of drugs into the lipid bilayer with less deformation of the cell membrane structure (Xue et al., 2019; Perini et al., 2020). Drug translocation, related to small later size, and the promising interaction with the biomembrane, suggested a possible role of GQDs as anti-TB adjuvant therapy that could enhance cell entry of several drugs used in TB management, known to show low permeability (Rey-Jurado et al., 2013; Hashemian et al., 2018).

Additionally, macrophages exposed to GQDs increase the presence of phosphoglycerides, sphingolipids, and oxidized lipids (Shao et al., 2023). While not completely elucidated, the role of host lipids and the changes associated with Mtb infection, play a key role in the pathogenesis of TB (De Maio et al., 2021). Indeed, we have recently demonstrated that phosphoglycerides such as phosphorylated phosphatidylinositol (PtdIns), cardiolipin (CL), and phosphatidic acid (PA), were recognized by a surface protein of *Mtb* which appears to ensure phosphate acquisition from the environment and mediate adhesion to host cell. Alternatively, sphingolipids accumulation in the eukaryotic membrane directly induces cell death (Lee et al., 2023). Additionally, the synthesis of sphingolipids, but not glycosphingolipids, is required for the phagocytosis of specific pathogens such as *Mtb*, thereby modulating actin dynamics and the formation of the phagocytic cup during engulfment and internalization (Lee et al., 2023). Hence, it will be important to investigate the consequences of GQDs on the homeostasis of lipids in infected macrophages.

*Mtb* has the potential to modulate macrophage polarization, with the balance of proinflammatory and anti-inflammatory macrophages driving the outcome of single granulomatous lesions (Mattila et al., 2013; Cadena et al., 2017). GQDs immunoregulatory properties are insufficiently investigated, especially in human primary immune cells (Habtamu et al., 2018), and certainly warrant detailed a conclusive investigation. Indeed, GQDs have been demonstrated to switch the polarization of macrophages from classically activated M1 to M2, enhancing intestinal infiltration of regulatory T cells and inhibiting Th1/Th17 polarization (Lee et al., 2020). T cell mediated immune responses of the Th1 type are widely regarded as protective against TB. The Th1 cytokine, interferon gamma (IFN-γ) is considered a key protective cytokine based on earlier studies, in both mice and humans, which showed that defects in the IFN-γ signaling pathways provoked higher susceptibility to Mtb infection and severe disease (Habtamu et al., 2018). Additionally, GQDs also reduced the numbers of interferon-γ-expressing T helper 1 cells, as well as the expression of Th1 transcription factor T-bet and proinflammatory cytokines TNF, IL-1, and GMSF (Tosic et al., 2019). While M1 polarized macrophages mediate inflammatory responses and increase the microbicidal activity, M2 polarized macrophages are related to persistent infection determining the escape of mycobacteria (Huang et al., 2015). Based on these findings, we shifted our experiments on human PBMCs that allow to investigate *Mtb* replication containment mediated by different cell types and in particularly the combination of both monocytes/macrophages and lymphocytes. Intriguingly, human PBMCs failed to control *Mtb* replication when treated with GQDs similarly to GO without affecting cell viability. We can speculate that non-toxic doses of GQDs may inhibit the production of proinflammatory and T helper 1 cytokines and augment the production of anti-inflammatory and Th2 cytokines by human PBMCs lead to uncontrolled *Mtb* replication (Tomic et al., 2017). Intriguingly, GQDs lowered the production of the target of rapamycin (mTOR), which correlated with the increase in autophagic flux in monocytes derived dendritic cells DC that could be another key point in failing *Mtb* replication control as we have demonstrated for other innovative anti-TB treatments (Bianco et al., 2023).

The emerging research and development in GQDs reveal their potential application in the biomedical field. Based on our observations, aminated GQDs when administrated with AMK appeared to increase the antimycobacterial drug efficacy, reducing the number of live mycobacteria. Our results elucidate the role of GQDs against *Mtb* infection, underlining its promising activity in macrophages but suggesting possible side effects on hPBMCs. These findings also suggest that beneficial effects in inflammatory T-cell mediated pathologies due to GQDs may be balanced with harmful in GQD-based anti-TB treatment as well as anti-cancer therapy.

## Materials and methods

### Characterization of GQDs

L-GQDs, NH_2_-GQDs and COOH-GQDs in aqueous solution were purchased from ACS Material. Optical density and fluorescence intensity spectra were obtained with a Cytation 3 Cell Imaging Multi-Mode Reader (Biotek). Both optical density and fluorescence intensity were recorded on samples diluted at 100 μg/mL as previously described (Papi et al., 2019; Perini et al., 2020, 2021, 2022). Fluorescence intensity was investigated by using excitation wavelengths from 250 to 520 nm with a step size of 10 nm and reading the emission from 300 to 700 nm. Fluorescence spectra were normalized to their corresponding maximum emission. Dynamic light scattering and zeta potential analyses were conducted using the Zetasizer Nano ZS (Malvern), equipped with a 633-nm He−Ne laser and operating at an angle of 173°. UV-transparent cuvettes were employed for experiments featuring a sample volume of 500 μL and a concentration of 100 μg/mL. Measurements were executed at a fixed position (4.65 mm) with an automatic attenuator. Each sample underwent three measurements, and the diffusion coefficient (D) was determined through cumulants’ analysis of autocorrelation functions. The equivalent hydrodynamic radius (Z-average size) was calculated using the Stokes−Einstein equation. Data analysis was conducted using the Malvern Zetasizer software. Chemical analysis of GQDs was conducted by using the spectrophotometer LUMOS II Bruker in ATR mode through Fourier transform infrared spectroscopy (FTIR). The specimen under examination was directly positioned on the crystal, and the resulting spectra were recorded within the wavenumber range of 4,000–600 cm^−1^.

#### Bacterial manipulation

Each experiment was performed by using the *Mycobacterium tuberculosis* (*Mtb*) reference strain *Mtb* H37Rv. Bacteria were grown in 7H9 broth medium (Difco) enriched with 10% albumin dextrose catalase (ADC) (Sigma-Aldrich) and 0.05% Tween 80 (Sigma-Aldrich), at 37°C and 110 rpm agitation, until an OD_600_ between 0.5 and 0.8 was reached. Bacterial cell culture was added with 20% sterile pure glycerol (Carlo Erba Reagents, Italy) and stored at −80°C. All experiments that involved *Mtb* manipulation were performed in Biosafety level 3 laboratory (BSL3) in the Institute of Microbiology of Fondazione Policlinico Universitario A. Gemelli (De Maio et al., 2021).

#### *In vitro* antimicrobial assay

To assess direct activity of GQDs, a suspension of *Mtb* H37Rv (10^5 CFUs/ml) was incubated alone or with GQDs Blue Luminescent (L-GQDs), Aminated GQDs (NH_2_-GQDs), or Carboxylated GQDs (COOH-GQDs) (ACS Material) at the final concentration of 50 μg/mL, or in combination with isoniazid (INH), amikacin (AMK) and linezolid (LZD) at the minimal inhibitory concentrations of 0.2 μg/mL, 1 μg/mL and 1 μg/mL, respectively (De Maio et al., 2019, 2020). Bacteria were incubated in 7H9 medium enriched with 10% ADC and 0.05% Tween80 at 37°C. Six and 24 h post incubation colony forming units (CFUs) were determined by plating serial dilutions of the suspensions on 7H11 solid medium (Difco) supplemented with 10% Oleic Albumin Dextrose Catalase (OADC) (Sigma-Aldrich) (Figure 2A). The antimicrobial assay with exponentially growing cultures was executed by incubating *Mtb* H37Rv (10^7 CFUs/ml) with non-functionalized L-GQDs and functionalized NH_2_-GQDs and COOH-GQDs alone or in combination with INH, AMK and LZD anti-mycobacterial drugs at the previously described conditions (Vilcheze et al., 2017). CFUs were determined at 0 (infection solution), 7, 10 and 21 days by plating serial dilutions of the suspensions on 7H11 solid medium (Difco) supplemented with 10% Oleic Albumin Dextrose Catalase (OADC) (Sigma-Aldrich) (Figure 3A).

#### Evaluation of cytotoxicity on eukaryotic cells

To measure cell viability under GQDs treatment, murine macrophages (J774 A.1 cell line) were cultured as described and then plated at the final concentration of 1.2 × 10^6 cell/ml in a 96-well plate. J774 cells were incubated overnight at standard atmosphere conditions (37°C and 5% CO_2_), until they were incubated with L-GQDs, NH_2_-GQDs and COOH-GQDs at concentrations of 50 μg/mL. Untreated cells were used as negative control, cells treated with 2% Triton X-100 as positive control. Triton X-100 interacts with lipid bilayers in a non-specific way, solubilizing bio-membranes and causing cell death (Koley and Bard, 2010; Mattei et al., 2017). Cells were incubated 24 h until lactate dehydrogenase (LDH) were measured, and cells were stained to assess monolayer integrity.

Briefly, LDH was evaluated on the supernatants of treated cells opportunely centrifuged to remove GQDs in the solution. Each supernatant was diluted before incubation with the substrate. Thirty minutes later, absorbance at 490 nm and 680 nm was measured using an automatic microplate reader, Cytation 5 Cell Imaging Multi-Mode Reader (Biotek Instruments). The percentage of cytotoxicity given by the LDH release is calculated as: $\%\mathrm{Cytotoxicity}=\frac{\mathrm{Compound}\;LDH\;\mathrm{activity}-\mathrm{Spontaneus}\;LDH\;\mathrm{activity}}{\mathrm{Maximum}\;LDH\;\mathrm{activity}-\mathrm{Spontaneus}\;LDH\;\mathrm{activity}}\;x\;100\text{;}$ where Spontaneus LDH activity is the value given by untreated cells and Maximum LDH activity is the value given by cells treated with Triton X-100. In the meanwhile, the cells were fixed with PFA 4% for 30 min and stained with crystal violet for 15 min. In another experimental setting the cells were plated and treated as previously described and the MTS Cell Proliferation Assay was performed to evaluate the cellular metabolic activity. 24 h post-incubation with GQDs, MTS reagent was added to each well and incubate for 0.5–4 h at 37°C. Absorbance was measured at 490 nm every half an hour (Figure 4A).

MTS assay was performed also for human immortalized hepatic (HepG2) and monkey renal (Vero) cells (Supplementary Figure 1), and for human Peripheral Blood Mononuclear Cells (hPBMCs) (Figure 6A), as previously described.

#### Macrophage cells culture and infection

Murine macrophages (J774 A.1 cell line) were cultured in Dulbecco’s modified Eagle’s medium (DMEM) (Euroclone) supplemented with 10% inactivated fetal bovine serum (FBS) (Euroclone, Italy), 1% L-glutamine (Euroclone) and 1% streptomycin–penicillin (Euroclone) and were incubated at 37°C and 5% CO2. Adherent cells were washed with sterile warm phosphate buffered saline (PBS) (Euroclone) and removed for experiments by using 1× trypsin in PBS (Euroclone). Cells were counted and re-suspended in DMEM supplemented with 2% FCS and 1% L-glutamine. Finally, cells were seeded in sterile 48 well plates (Euroclone) at a concentration of 1.2 × 10^6 cells/ml and incubated overnight until infection or treatment. J774 cells were infected with *Mtb*, with multiplicity of infection (MOI) of 1 (1 bacterium to 1 cell), resuspended in the cell culture medium (De Maio et al., 2014). One hour post infection, infected cells were washed with sterile warm PBS to remove extracellular bacteria and treated with GDQs for 1 day. Finally, INH, AMK and LZD were administrated at the concentrations above indicated. Cells were incubated in standard atmosphere conditions for 24 h when CFUs were assessed by harvesting cell monolayer with sterile 0.1 mL of sterile 0.05% Triton X-100 (Sigma-Aldrich). Serial dilutions were carried out before plating on 7H11, containing 10% OADC. Plates were then incubated at 37°C for 15 days (Figure 5A).

#### Evaluation of the cytotoxicity on peripheral whole blood cells

Peripheral whole blood collected by heathy volunteers and was treated with L-GQDs, NH_2_-GQD and COOH-GQD at the final concentrations of 50 μg/mL for 1 h at 37°C. Untreated whole blood was included as a control (Figure 6A). Cell populations were identified using a combination of 8 fluorochrome-labeled monoclonal antibodies: HLA-DR V450 (clone L243), CD45 BV500 (clone HI30), CD64 FITC (clone 10. 1), CD4 PE (clone RPA-T4), 7-Amino-Actinomycin D (7-AAD) PerCP-Cy5.5, CD3 APC-H7 (clone SK7) (BD Biosciences), CD8 PC7 (clone SFCI21Thy2D3), and CD25 APC (clone B1.49.9) (Beckman Coulter). From each suspension, an aliquot was collected and incubated with the previously described antibodies for 20 min at room temperature. Cells were washed with PBS containing 1% bovine serum albumin (BSA), by centrifugated at 1500 RPM for 5 min and finally resuspended in PBS. Data were acquired using the Cytoflex cytofluorimeter and analyzed using Kaluza software (Beckman Coulter) (Salustri et al., 2023). Data analysis was carried out following a gating strategy composed of several and serial steps on at least 20,000 events. 7AAD PC5.5-A versus Side Scatter (SSC-A), Forward Scatter Area (FSC-A) versus SSC-A, and Forward scatter Height (FSC-H) versus FSC-A gatings were achieved to distinguish live cells from cell debris, artifacts and GQDs aggregates, by size and complexity. The following gatings were performed to distinguish cell populations: (a) CD3 APC-A750-A versus SSC-A was used to select lymphocyte population, (b) CD8 PC7-A versus CD4 PE-A was used to distinguish CD8 from CD4 lymphocyte populations; (c) a CD64 FITC-A versus SSC-A was used to evidence monocytes. Cell activation and maturation rates were analyzed using DR PB450-A versus CD25 APC-A and CD64 FITC-A versus DR PB450-A were used to evaluate lymphocytes and monocytes activation, respectively.

#### Human peripheral blood mononuclear cells isolation and infection

Human Peripheral Blood Mononuclear Cells (hPBMCs) were isolated from buffy coats of healthy volunteers (De Maio et al., 2021; Bianco et al., 2023). Healthy donors were recruited among people who had recently tested negative for QFT negative, not vaccinated with BCG, male, Caucasian, and aged between 30 and 35 years. Written informed consent was obtained from each subject involved in the study donor (ID:3715/2021). The study was conducted in accordance with the Declaration of Helsinki and approved by the Institutional Review Board (or Ethics Committee) of Policlinico A. Gemelli for studies involving humans. Briefly, blood was diluted with sterile PBS (1:1) and gently poured into a tube containing Ficoll Human Lympholyte^®^ (CEDARLANE, Ontario, Canada). Following a centrifugation at 1500 RPM for 30 min at room temperature (23°C) without brake. Lymphocytes and monocytes were collected and washed with PBS. Finally, cells were resuspended in Roswell Park Memorial Institute (RPMI) 1,640 culture medium (Euroclone) enriched with 10% fetal bovine serum (Corning), 1% Glutamine (Euroclone), 1% Sodium Pyruvate (Euroclone) and plated in 48 well plate (NEST) at a final concentration of 1.2 × 10^6 cells/ml (De Maio et al., 2021). hPBMCs were infected with a Multiplicity of Infection (MOI) of 1 respect to monocytes (corresponding to ~5% of the hPBMCs, ~ 6×10^4 cells/ml). 24 h post infection hPBMCs were treated with L-GQDs, NH_2_-GQDs and COOH-GQDs. The next day, drugs were administrated at the previously indicated MIC concentration. Bacterial survival was evaluated at 4 and 8 days post infection by CFUs counting (Figure 7A).

### Statistical analysis

Data were collected and organized using Microsoft Excel software version 16.69.1 and were analyzed by using GraphPad Prism software version 9.0.0 (GraphPad software) and R v4.0.2. All experiments were performed in scientific duplicates and technical triplicates. All data were expressed as mean plus SD and analyzed by two-way ANOVA followed by the appropriate correction.

## Data availability statement

The original contributions presented in the study are included in the article/Supplementary material, further inquiries can be directed to the corresponding author.

## Ethics statement

The studies involving humans were approved by Institutional Review Board (or Ethics Committee) of Policlinico A. Gemelli for studies involving humans. The studies were conducted in accordance with the local legislation and institutional requirements. The participants provided their written informed consent to participate in this study.

## Author contributions

GS: Writing – review & editing. GP: Writing – review & editing. AS: Writing – review & editing. IP: Writing – review & editing. RR: Writing – review & editing. VP: Writing – review & editing. CI: Writing – review & editing. SB: Writing – review & editing. MiS: Writing – review & editing. MaS: Writing – review & editing. MSp: Writing – review & editing. MP: Formal analysis, Resources, Supervision, Writing – original draft, Writing – review & editing. GD: Writing – original draft, Writing – review & editing. FM: Writing – original draft, Writing – review & editing.

## Supplementary material

SUPPLEMENTARY FIGURE S1 Assessment of GQDs’ cytotoxicity on hepatic and renal immortalized cells. Hepatic immortalized cells (HepG2) and renal immortalized cells (Vero) were seeded in a 96 well plate. When 90% cell monolayer was reached, cells were treated with 50 μg/ml GQDs. Untreated cells and cells treated with 2% Triton X-100 were used as negative and positive controls, respectively. The next day, MTS assay was performed to evaluate cellular metabolic activity both for hepatic cells **(A)** and for renal cells **(B)**.
