# Supplementary figures and images for: Unraveling the potential of graphene quantum dots against Mycobacterium tuberculosis infection
Source: Front Microbiol. 2024 May 7;15:1395815. doi: 10.3389/fmicb.2024.1395815 (PMC11107295; doi:10.3389/fmicb.2024.1395815)

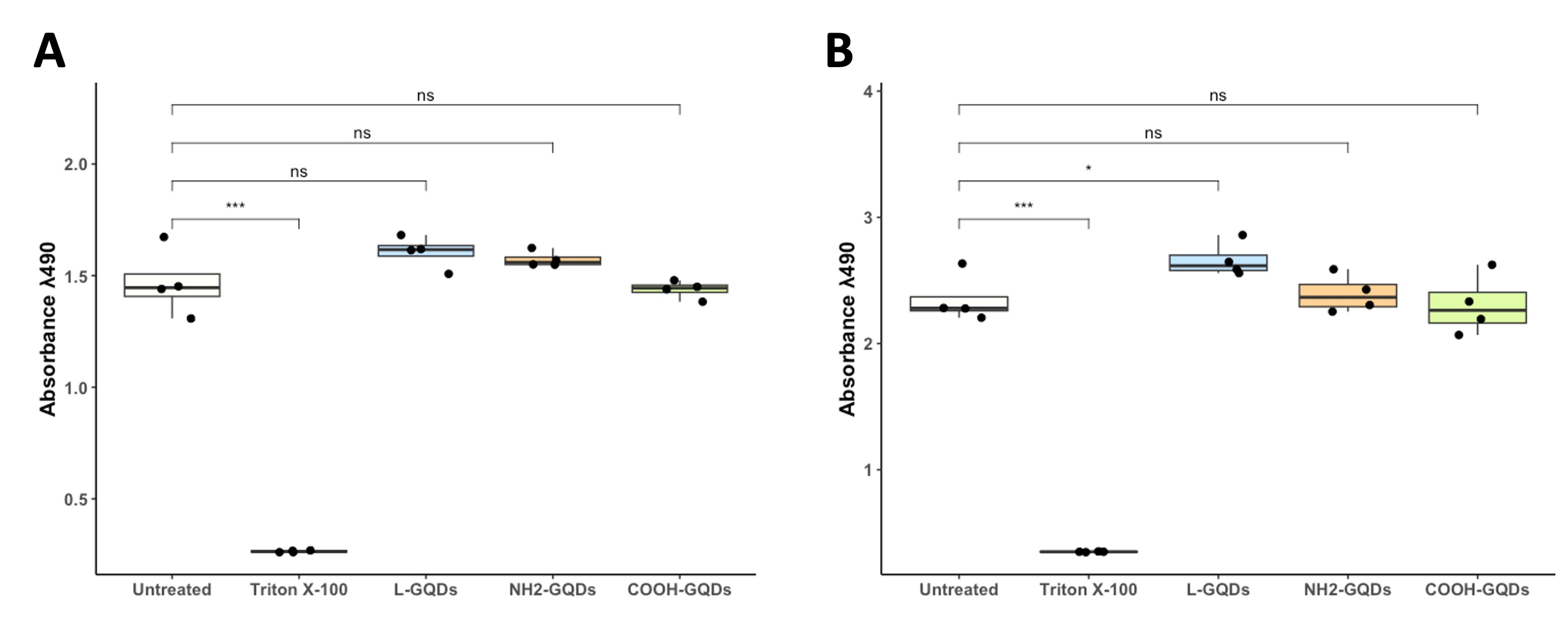

Supplement: SUPPLEMENTARY FIGURE S1 — Assessment of GQDs’ cytotoxicity on hepatic and renal immortalized cells. Hepatic immortalized cells (HepG2) and renal immortalized cells (Vero) were seeded in a 96 well plate. When 90% cell monolayer was reached, cells were treated with 50 μg/ml GQDs. Untreated cells and cells treated with 2% Triton X-100 were used as negative and positive controls, respectively. The next day, MTS assay was performed to evaluate cellular metabolic activity both for hepatic cells (A) and for renal cells (B). [file Image_1.jpg]
